# Who owns (or controls) health data?

**DOI:** 10.1038/s41597-024-02982-1

**Published:** 2024-02-01

**Authors:** Scott D. Kahn, Sharon F. Terry

**Affiliations:** 1 LunaPBC, San Diego, California 92121 USA; 2 Genetic Alliance, Damascus, Maryland 20762 USA

**Keywords:** Paediatric research, Clinical trial design

## Abstract

The ongoing debate on secondary use of health data for research has been renewed by the passage of comprehensive data privacy laws that shift control from institutions back to the individuals on whom the data was collected. Rights-based data privacy laws, while lauded by individuals, are viewed as problematic for the researcher due to the distributed nature of data control. Efforts such as the European Health Data Space initiative seek to build a new mechanism for secondary use that erodes individual control in favor of broader secondary use for beneficial health research. Health information sharing platforms do exist that embrace rights-based data privacy while simultaneously providing a rich research environment for secondary data use. The benefits of embracing rights-based data privacy to promote transparency of data use along with control of one’s participation builds the trust necessary for more inclusive/diverse/representative clinical research.

## Introduction

There is a decades-old practice of so-called de-identifying health data so the information could be shared openly for secondary use in research^1^. The process of deidentifying includes removing directly identifying data such as name and birthdate, and removal of indirect identifiers that in aggregate increase the risk of re-identification. As computing power has increased exponentially, so has the development of machine learning (ML) and artificial intelligence (AI) algorithms that can process collections of such de-identified data to re-identify individuals^2–13^. Such risks will vary with different data types making the assessment of this risk important prior to data release and making the interpretation of “de-identified” data under HIPAA more nuanced. With the risk of re-identification as a present-day reality, involving individuals in sharing their health data for research is critical, especially regarding transparency around who is performing the research and for what purpose. One powerful framework to achieve these objectives centers on rights-based data privacy regulations that assert the control of the use of data collected about an individual rests with the individual rather than with the institution that collected the data^14^.

A family of rights-based data privacy regulations has been inspired by the European Union’s implementation of the General Data Protection Regulation (GDPR) in 2018^15^. GDPR establishes data protection as a basic human right by acknowledging that all data collected on an individual can present risks to the individual (e.g., re-identification, reputational risks, etc.) and that the individual has the right to control the use of such data. GDPR does not define direct or indirect identifiers that must be removed, rather it considers all information collected on an individual as a pseudonymous record of data that can be evaluated for risk to the individual. Data that is evaluated as “low risk” to the individual is defined as anonymous data and can be freely shared for research and for other purposes. These more precise definitions of data are not part of the common vernacular; “de-identified data” is simply a type of pseudonymous data in which the risk to the individual has been reduced by removing directly identifying data types.

Focusing on health data, it should be apparent that removing directly and indirectly identifying data types does not fully dissociate the data from the individual; the data remains personal data and should be handled as such especially when the risks of re-association are more likely. And while the ownership of data collected on an individual by a healthcare provider for the practice of medicine may be debated^16^, the control of these data for secondary research use should rest firmly with the individual (or their parent, guardian, or similar). This distinction is central to the tenets of data protection as a human right, and it presents several challenges to the governance of data and the management of informed consent. Whereas in the past, blanket consent could be sought for all possible (research) uses, it is impossible to provide the necessary information to enable an individual to make such a decision, especially when they are seeking care. Moreover, much health-related data today is collected outside of a healthcare environment (e.g., apps, wearables, etc.) that are not bound by healthcare regulations but are still subject to data protection regulations. With the emphasis of real-world data and patient reported outcomes it seems prudent to include these non-clinical data types in any discussion of ownership and/or control of use.

## Background

A solution to the apparent incompatibility of open-ended research and distributed control of shared data can be found in rights-based data privacy frameworks as an enabler of more inclusive data aggregations rather than being an impediment to research efforts. Building a data sharing and analysis platform with privacy-by-design^17^ at its architectural foundation has been accomplished^18^. It yields a platform that provides a research environment that becomes familiar to the participant with several useful enhancements.

Obtaining informed consent with a backdrop of purpose limitation requires a relationship with study participants resulting in ongoing engagement of each participant in the study objectives. When this is achieved, it can be straightforward to request consent for new studies and study objectives; no researcher can foresee all the possible changes to a study’s direction as data is collected, analyzed, and new lines of inquiry are made apparent. Robust informed consent management and governance must become a key capability of a privacy-based research platform. In a similar vein, data minimization requires parsimony in data collection. As with incremental informed consent, new data can be requested as study questions evolve and as consent is received^19,20^.

Once assembled, engaged study cohorts persist and can be re-approached for new studies through a request for additional or new informed consent. Persistence of the cohort supports studies of outcomes longitudinally part of or downstream of a study protocol – a more comprehensive approach to “post-market surveillance” that can extend to years and decades if there is perceived informational benefit. As an example, gene therapy approvals generally require long term follow-up. For therapies, these persistent cohorts offer a direct path to follow-on therapies to address unmet medical needs or to improve upon observed counterindications. And as studies are initiated with a growing number of persistent cohorts, it is reasonable to speculate on learnings that might be gleaned from observational studies that span multiple conditions and that hopefully span an inclusive set of ethnicities participating^21,22^.

The ability to interact with a study cohort without introducing bias can be directly solved through a recontact facility that can identify “pools”: individuals identified by attribute(s) rather than by personally identifying information (PII). For example, renewing a request to complete a survey instrument, or to share some type of pre-existing health information can be accomplished at a group level and without any PII through a platform capability that separates email or texting information from the study. And while this might also be accomplished by a contract research organization (CRO) that is managing a blinded trial, this introduces a human element that could be a source of PII leakage. It also introduces another actor that could confuse participants as to who is responsible for the study. Providing direct access to a study’s administrator increases efficiency in the process and supports a more dynamic approach to cohort interactions than traditional CROs can offer.

Using a privacy-by-design platform built to implement rights-based data privacy offers a unique path to data reuse that respects an individual’s right to be informed on additional research uses of their shared personal data. Rather than asking study participants to grant unbounded consent for the use of their personal data – which can be difficult to provide the contextual information around which consent can be granted – informed consent can be sought dynamically and with a precise context to guide the individual. In contrast to the challenging process of re-consenting a cohort, dynamic consent supports a modern solution to privacy regulations around data minimization and informed consent requirements.

It is worth reiterating that de-identification of health data, as is performed to comply with HIPAA, does not necessarily render the data non-personal data under data protection regulations. Moreover, “de-identified” data can still be used to re-identify an individual using AI/ML methods^2–13^. So-called pseudonymous data devoid of HIPAA identifiers should be treated like personal data for which informed consent is obtained. When the data is not directly collected from an individual, such as synthetic data or when random noise is used to alter the data from its source from an individual, it can be considered anonymous and not covered by privacy regulations.

A very important aspect of working with a privacy-by-design platform is the ability to return study data to study participants that may be useful in managing their health journeys outside the study. For example, consider the case where genomic data is collected as part of the study. Here, these data can be returned to each individual for subsequent use outside of the study to guide therapy selection in the case of disease, or the proactive use of genomic data to manage health and prevent disease^23^. Both uses of returned data are at the vanguard of medicine. They have enormous unrealized potential, and researchers worldwide are actively working to piece together the interconnections between one’s genomics and their health and disease outcomes. In short, as precision medicine continues to develop, such return data will increasingly be useful to healthcare providers and to researchers seeking to further understand and mitigate disease.

## Discussion

There are historical^24–26^ and ongoing examples^27,28^ of health studies that have eroded participant trust and consequentially contributed to the lack of inclusion by under-represented groups in clinical research. And while there are benefits of diverse inclusion for the discovery of health tenets, overcoming issues of mistrust are a persistent barrier to resolution. The previous work^21,22^ to understand how to resolve concerns around trust highlights the need for data use transparency^29^ and to guarantee equity around any financial benefits that might flow from the research performed^18^. Transparency can be straightforwardly addressed by ensuring that each participant in a study always maintains control of the use of their data through their informed consent. Rights-based data privacy laws such as GDPR and the California Privacy Rights Act (CPRA)^30^ codify this control through a right to purpose limitation (i.e., the researcher must be concisely clear on the intent of the study) and a right to revoke one’s consent and remove one’s data if a study diverges from the stated objectives and/or the study no longer is consistent with an individual’s values. Ironically, rather than being an impediment to research, the reliance on informed consent in conjunction with purpose limitation via data privacy laws and guidelines can more appropriately be seen as enablers of inclusive research by reducing the risk of data misuse for individuals that would otherwise have this as a primary concern (e.g., re-use for immigration enforcement, etc.).

The global experience with the COVID-19 pandemic has hastened the adoption of distributed clinical trials (DCT) that benefit from Real World Data (RWD) and Patient Reported Outcome (PRO) data. RWD and PRO allow the inclusion of lived experiences into study design, and distributed trials support participation with more convenience for participants that have digital access, which in turn supports participation by groups that would otherwise lack the means to be represented. Lacking digital access, barriers to participation persist. Now that many of the restrictions around COVID-19 containment are lifted, the advantages of DCTs can be explored and developed to harness their advantages, especially regarding group inclusion.

Another benefit of clinical studies that are distributed, inclusive, and whose data is managed using a rights-based data privacy framework is that studies can persist even after initial study objectives have been achieved. For example, for studies focused on the characterization of health outcomes from a novel therapeutic or therapy, there could be enormous benefit in revisiting the cohort to understand outcome progression many years after the trial has concluded in a more comprehensive and possibly opportunistic manner than simply tracking adverse event reporting. Having many such persistent trial cohorts also provides an opportunity to understand therapies and interventions comparatively to guide usage and even studies of health economics that capture longitudinal co-morbidities.

An unstated assumption is that individuals who can control their data use are also more engaged in the use of their data. Promoting individuals from study subjects to study participants or even study partners is a different way to calibrate patient-centered research and patient engagement^31–33^. It is also sometimes explicitly said, or implicated, that decentralized participants will be unable or will not consent to participate, thus skewing research cohorts. This should not negate the critical autonomy of participants in research.

Finally, we have tried to highlight that in the contemporary era of nearly limitless computational power coupled to advances in AI and ML that health data can never be completely de-identified. This is embraced within rights-based data privacy frameworks by the characterization of risks to the ***individual*** through data impact assessments. These risk assessments on behalf of the individuals whose data is being studied is a tectonic shift towards honoring the rights of individuals over the institutional models of control that define principal investigator-driven research that is *de rigueur* in clinical research today.

## Summary

The topic of who owns health data and who should control the secondary use of health data is both complex and subject to the laws under which the data was collected, and the citizenship of the individual on whom the data was collected. We argue that the control of one’s health data for secondary research use is of highest concern since this extends well beyond the context in which the data was collected in the first place^34,35^. It has been argued that de-identification is a misnomer in a world of advanced AI/ML methods, and that the global move to embrace individual data privacy rights (i.e., via data protection regulations) requires a rethinking of data collection and informed consent processes currently in place. And while such changes require infrastructural changes, embracing individual privacy rights offers a path to enhanced participant inclusion and engagement; engaged “participants” have a far greater value for research than enrolled study “subjects”.

Changes to the handling of health data for secondary use can also usher in new capabilities beyond better trial inclusiveness. The adoption of remote collection and interaction necessary for participant engagement also supports distributed clinical trial models that facilitate the inclusion of lived experience and social determinants of health to be accounted for in trial design. And a consequence of such distributed trial designs that lever digital data collection and more engaged participants are that study cohorts can persist post trial for observational studies that span multiple conditions and that span more inclusive ethnic representation.

## Data Availability

No data is directly associated with this article.
